# Occurrence of *Listeria monocytogenes* in Ready-to-Eat Meat Products and Meat Processing Plants in Spain

**DOI:** 10.3390/foods4030271

**Published:** 2015-07-14

**Authors:** Diego Gómez, Laura Pilar Iguácel, Mª Carmen Rota, Juan José Carramiñana, Agustín Ariño, Javier Yangüela

**Affiliations:** 1 Department of Animal Production and Food Science, Veterinary Faculty, University of Zaragoza, c/Miguel Servet 177, Zaragoza 50013, Spain; E-Mails: dgl.15@hotmail.com (D.G.); crota@unizar.es (M.C.R.); carramin@unizar.es (J.J.C.); jyangu@unizar.es (J.Y.); 2 Agrifood Research and Technology Centre of Aragón (CITA), Avda. Montañana 930, Zaragoza 50059, Spain; E-Mail: lpiguacel@cita-aragon.es

**Keywords:** *Listeria monocytogenes*, occurrence, ready-to-eat meat products, work surfaces, meat processing plant

## Abstract

The aim of this work was to study the occurrence of *Listeria monocytogenes* in several types of ready-to-eat (RTE) meat products and in the environment of meat processing plants. A total of 129 samples of RTE meat products and 110 samples from work surfaces and equipment were analyzed. *L. monocytogenes* was detected in 6 out of 35 cooked products (17.14%), 21 out of 57 raw-cured products (36.84%), and 9 out of 37 dry-cured, salted products (24.32%). The number of sample units that exceeded the food safety limit of 100 cfu/g decreased from the manufacture date to half shelf life, and then it was further reduced at the end of shelf life. *L. monocytogenes* was detected in 25 out of 110 (22.72%) food contact surfaces. The number of positive and negative results from both food and environmental samples were cross-tabulated and the calculated Cohen’s kappa coefficient (κ) was 0.3233, indicating a fair agreement in terms of *Listeria* contamination. *L.* *monocytogenes* was recovered after cleaning and disinfection procedures in four plants, highlighting the importance of thorough cleaning and disinfection.

## 1. Introduction

*L. monocytogenes* has been isolated from a wide variety of ready-to-eat (RTE) foods and is responsible for several outbreaks of listeriosis linked to the consumption of meat, poultry, dairy, fish and vegetable products. Even when *L. monocytogenes* is initially present in small quantities in a foodstuff, it can multiply at varying rates during chilled storage depending on the type of food product, both under aerobic and anaerobic conditions, adapt to disinfectants and adhere to various surfaces [1]. The ability of *L. monocytogenes* to multiply in various foods at temperatures as low as 2 to 4 °C makes the occurrence of *L. monocytogenes* in ready-to-eat (RTE) foods, such as cooked, raw-cured and dry-cured salted meat products, of particular concern [2,3,4].

Even though primary contamination can occur, the frequency and level of *L. monocytogenes* in RTE meat products are mostly related either to recontamination of the product before its final packaging or to later handling, during its commercialization or use in the home [2]. It is well known that working environments, equipment and surfaces that come into contact with food are common sources of cross-contamination [5]. Food-contact surfaces can represent a significant hazard, especially if microbial aggregates, known as biofilms, have formed attached to the work surfaces [6]. *L. monocytogenes* has biofilm forming ability, which could be an important cause for its persistence in food processing environments [1]. In fact, many studies have shown that *L. monocytogenes* is able to adhere to and form biofilms on surfaces in contact with food such as polyethylene, polyvinyl chloride, glass and stainless steel [7,8].

There are currently no sufficiently effective, fast protocols to reveal the presence of microorganisms, their adhesion to and possible multiplication on work surfaces in a routine way, or to assess the effectiveness of any disinfectant applied. Sampling techniques for environmental surfaces according to ISO 18593:2004 include direct contact plates, swabs, sterile pre-moistened towel, and sponge. However, the detection ability and repeatability of environmental sampling procedures are influenced by the time of sampling, the state of the surface, the surface type, the sampling device, and the skill of the operator, especially when the cell concentration on the surface is low [9]. Recently, a new mini-roller sampling device for the efficient recovery of *L. monocytogenes* from food contact surfaces has been described and tested in the present authors’ laboratory [10].

The objective of this work was to study the occurrence of *L. monocytogenes* in several types of RTE meat products (cooked, raw-cured, and dry-cured and salted) and in the environment of several meat processing industries from across Spain. The surveyed foods were RTE and therefore intended to be consumed without any further heat treatment.

## 2. Experimental Section

### 2.1. Sampling of RTE Meat Products

A total of 129 samples of RTE meat products were taken during 2012 and 2013 of which 87 were taken at manufacturing from meat industries of six Spanish provinces and 42 were purchased at retail in Zaragoza. Additionally, 110 environmental samples were taken from the same manufacturing plants. Table 1 shows the number of samples of RTE meat products and work surfaces from each of the 32 sampled meat processing plants. The RTE meat products can be grouped into three types: Cooked meat products (35 samples, mostly Frankfurters), raw-cured sausages (57 samples, mostly “*chorizo*” and “*salchichón*” type), and sliced dry-cured salted ham (37 samples).

**Table 1:** Number of samples (*n*) of ready-to-eat (RTE) meat products and work surfaces from each meat processing plant.

Meat Processing Plant	Province	Samples (*n*) of RTE Meat Products			Samples (*n*) of Work Surface Samples				
		Cooked	Raw-Cured	Dry-Cured, Salted	Stainless Steel		Conveyor Belt		Cutting Board
					Dirty	Clean	Dirty	Clean	Dirty
1	Zaragoza	4	3		1	3			
2	Zaragoza	2	2		2				
3	Zaragoza		2	2	4				1
4	Zaragoza		2		6	1			
5	Zaragoza			4	5		3		
6	Teruel			2	3	1	4	2	
7	Teruel	2			1		2		
8	Teruel		1	1	1		2		
9	Teruel			5	4	1	4	2	
10	Teruel			1	1		2		
11	Teruel		1	1	1		2	1	
12	Teruel			2	3		4		
13	Salamanca		2	1		2			
14 *	Salamanca		2						
15	Salamanca	1	2		1	2			
16	Salamanca		2			2			1
17	Salamanca		2		2	1			
18	Salamanca		2		1	1			
19	Cáceres		3	1	2	3			
20	Cáceres	1	2		1				
21	Cáceres		2	1	1				
22	Cáceres		1	1	1	1			
23	Badajoz		2			2			
24	Badajoz		2		2	2			
25 *	Badajoz			1					
26	Badajoz	1	1		1	1			
27	Badajoz		3		1				
28	Huelva	1	5		2	1			
29	Huelva	1	1		1	1			
30	Huelva		2			2			
31	Huelva	1	2		2	1			
32	Huelva	1				1			1
Total	--	15	49	23	50	29	23	5	3

* In plants 14 and 25 it was not possible to take work surface samples.

Regarding food categories, Commission Regulation (EC) No 2073/2005 indicates: (i) ready-to-eat foods able to support the growth of *L. monocytogenes*; and (ii) ready-to-eat foods unable to support the growth of *L. monocytogenes*. The food safety limit for both RTE foods is the same (100 cfu/g) when placed on the market during their shelf-life. However, for RTE to be able to support the growth of *L. monocytogenes* before the food has left the immediate control of the food business operator who has produced it, the limit is absence in 25 g. In the present study, cooked products belong to category a, while raw-cured and dry-cured salted products belong to category b. For convenience, and considering the general objective to keep the concentration of *L. monocytogenes* in food below 100 cfu/g, a single food safety limit of 100 cfu/g was applied.

Then, to determine the number of sample units that exceeded the food safety limit of 100 cfu of *L. monocytogenes*/g during product shelf life, five packaged samples of each RTE meat product were taken, all belonging to the same production batch. The first sample was analyzed on the same sampling day (day 0, that is the day of manufacturing in the plant or the day of purchase at retail establishment), and the remaining were stored at 4 and 10 °C and sampled at half and at the end of shelf-life as indicated in the label by the manufacturer.

### 2.2. Sampling of Work Surfaces

Thirty-two meat processing plants located in Zaragoza (5), Teruel (7), Salamanca (6), Cáceres (4), Badajoz (5) and Huelva (5), were visited during 2012 and 2013 and work surfaces were sampled (see Table 1). These environmental samples were directly taken from food-contact surfaces such as machines, knives, blades, tables, cutting boards, containers, and conveyor belts. All sampling was done with 100% wool fiber mini-roller as previously described [10], using 100 cm^2^ sterile stainless steel templates where possible (flat and smooth surfaces). In total, 110 surface samples were taken (61 were 100 cm^2^ and 49 for other smaller size): 71.8% of the samples were taken on stainless steel, 25.5% from polyvinyl chloride conveyor belts, and 2.7% from high molecular weight polyethylene cutting boards. In each processing plant the sampling of work surfaces was made (i) during manufacturing of sampled RTE meat products (so called “dirty” sites); and (ii) after cleaning of the equipment surfaces (called “clean” sites).

### 2.3. Microbiological Analysis

The detection of *L. monocytogenes* was investigated in all of the samples taken, both from RTE meat products and work surfaces. In addition, enumeration of *L. monocytogenes* was made in all the RTE meat product samples and in the 61 100 cm^2^ surface samples. In all cases, the samples were analyzed within a maximum of 12 h from being taken, and were stored at 4 °C during this period. The method followed was based on ISO 11290-1:1996/AM1:2004 and ISO 11290-2:1998/AM1:2004. For *Listeria* detection, the method consisted of a double enrichment in half Fraser and Fraser selective broths. The initial incubation in half Fraser broth was carried out for 24 h at 30 °C. The second step of the enrichment was carried out in Fraser broth for 48 h at a temperature of 37 °C. Half Fraser broth contains half the concentration of nalidixic acid and acriflavin of that found in Fraser broth. Cultures obtained in half Fraser and Fraser broths were plated on ALOA agar in triplicate. After incubation at 37 °C for 24–48 h, the colonies of presumptive *L. monocytogenes* were subcultured and confirmed by means of appropriate morphological and biochemical tests (β-hemolysis and rhamnose, xylose, and mannitol fermentation) and identified using the API^®^ Listeria gallery. The limit of sensitivity for the detection of *L. monocytogenes* was one cell in 25 g samples (*i.e.*, 0.04/g).

For the enumeration of *Listeria*, the test portion was decimally diluted in half Fraser broth base without selective agents, homogenized and kept at room temperature for one hour to ensure the adaptation of *Listeria*. Then, 0.333 mL of the homogenate was surface-plated on dishes of ALOA agar in triplicate, and incubated at 37 °C during 24–48 h. The colonies of presumptive *L. monocytogenes* were counted, sub-cultured and identified as described above. Calculation of the *L. monocytogenes* contamination level is carried out according to the number of confirmed colonies and expressed as *L. monocytogenes* cfu/g. The limit of sensitivity for the enumeration of *L. monocytogenes* was 10 cfu/g.

### 2.4. Statistical Study

The Cohen’s kappa statistic [11] was applied in order to study the agreement between the number of positive and negative results from both RTE meat products and environmental samples from each meat processing plant in terms of *L. monocytogenes* contamination.

## 3. Results

### 3.1. Listeria Monocytogenes in RTE Meat Products

*Listeria monocytogenes* was detected on the arrival at the laboratory (day 0) in 6 out of 35 RTE cooked products (17.14%), 21 out of 57 RTE raw-cured products (36.84%), and 9 out of 37 RTE dry-cured, salted products (24.32%). On average, *L. monocytogenes* was detected in 27.90% of RTE meat products during manufacturing (day 0) or at retail (day of purchase). The occurrence during storage showed that the percentage of positive samples of RTE cooked products remained almost unchanged at 4 °C (17.14% at half shelf-life and 14.29% at the end of shelf-life), and it was somewhat lower at 10 °C (11.43 and 5.71% at half and at the expiry date, respectively). The *L. monocytogenes* percent positives in RTE raw-cured products stored at 4 °C were 31.58% at half shelf-life and 22.81% at the end of shelf-life, while at 10 °C the percent of positives was 15.79 and 5.26% at half and end of shelf-life, respectively. However, most noticeable changes were observed in RTE dry-cured, salted product where percent positives were 24.32% at day 0, while only one positive sample (2.70%) was detected at either 4 or 10 °C throughout the whole shelf-life of the product.

Most samples of RTE products showed counts of *L. monocytogenes* below 100 cfu/g at the beginning of the storage. Thus, *L. monocytogenes* counts were determined at day 0 in three cooked samples (10–820 cfu/g), 11 raw-cured samples (10–910 cfu/g) and two dry-cured salted samples (10 cfu/g). Despite the relatively high prevalence of *L. monocytogenes*, only one sample of raw-cured sausage contained this pathogen at 190 cfu/g at half shelf-life, a level exceeding the food safety limit of 100 cfu/g. Enumeration of *L. monocytogenes* apparently decreased during storage at 4 °C and 10 °C, and at the end of shelf life counts were only possible in three samples (20–40 cfu/g), the remaining being <10 cfu/g.

### 3.2. Listeria Monocytogenes in Environmental Samples from Meat Processing Plants

*L. monocytogenes* was detected in 25 out of 110 (22.72%) environmental samples from meat processing plants, which is another example of the high persistence of this pathogen in environmental samples from food processing plants. Seventeen (17) positive samples came from stainless steel surfaces, six from conveyor belts, and two from cutting boards. As expected, the percentage of positive samples was much higher from dirty surfaces (84%) than from clean surfaces (16%). *L. monocytogenes* was recovered after cleaning and disinfection procedures in four processing plants, highlighting the importance of thorough cleaning and disinfection procedures. Enumeration of *L. monocytogenes* was achieved in only two samples, a stainless steel table (49 cfu/cm^2^) and the hopper of a sausage stuffing machine (1 cfu/ cm^2^).

Despite no strain characterization was carried out in this study, the *Listeria* occurrence in RTE meat products and in environmental samples could be used to compare both contamination scenarios. With this caution in mind, the results of *L. monocytogenes* occurrence in both food and in environmental samples were cross-tabulated to determine if there was a difference between the number of samples in which the pathogen was detected (or not detected) in both sample types *vs.* the number of samples in which the pathogen was only detected in one sample type. Cohen’s kappa statistic was used as it is a summary measure of cross-classification that allows for the agreement expected by chance. The calculated Cohen’s kappa coefficient (κ) was 0.3233, indicating a fair agreement [11].

## 4. Discussion

The study of the prevalence of *L. monocytogenes* in RTE foods and in the processing environment is very important. Ready-to-eat meat products may be contaminated by *L. monocytogenes* at several stages such as the raw materials (meat), during the production process (equipment) or by contact with contaminated unprocessed raw materials, unclean surfaces or people. Once introduced into the plants, *L. monocytogenes* can persist over time in the processing environment. Not surprisingly, *L.* *monocytogenes* is among the most frequently detected pathogens in meat products, and several studies have documented the incidence of the pathogen reaching prevalence levels of up to 40%–45% [1].

Results in current study revealed a higher prevalence of *L. monocytogenes* in RTE meat products compared with other authors that conducted a two-year longitudinal study on 1744 samples from RTE producing industries in the US, including sponge samples from surfaces either in contact or not with foodstuffs as well as finished RTE products [4]. They observed a *L. monocytogenes* incidence ranging between 1.7% and 10.8% in the first year and between 0.9% and 8.7% in the second. Although there are studies with higher percentages such as that carried out in Brazil by Araujo *et al.* [12], which reported a *L. monocytogenes* incidence of 60.0% in meat products based on sliced turkey meat. Table 2 reports some occurrence studies of *L. monocytogenes* in RTE meat products.

In general, a common pattern in the positive presence of *L. monocytogenes* was observed, consisting of a relatively high prevalence at day 0 (27.9% on average), followed by somewhat lower prevalence during storage at 4 °C (14.7%) and 10 °C (4.7%). Martins and Leal [13] carried out a similar study, analyzing 130 samples of salami at two stages during shelf-life. Positivity of *L. monocytogenes* at early stage was 30.8% and decreased to 18.5% at late shelf-life stage. In a study on inoculated RTE meat products, Ingham *et al.* [14] reported that numbers of *L. monocytogenes* fell for all products during storage, ranging from a decrease of 0.8 log CFU on smoked cured beef slices during 11 weeks under vacuum at 5 °C to a decrease of 3.3 log CFU on a pork rind product stored 5 weeks under air at 21 °C.

It is well known that *L. monocytogenes* can have sporadic occurrence and uneven distribution on food influenced by the physicochemical characteristics of the products (pH, salt, aw, additives, competitive microbiota), the storage temperature as well as the intrinsic strain characteristics. Thus, the lower *Listeria* incidence towards the end of shelf life revealed in the present study could partly be explained by the theory of metabolic exhaustion and stress response in hurdle technology applied to the manufacture and storage of RTE meat products [15]. For many RTE meat products, the reduction of water activity through the addition of salt and cooking or drying, or the reduction of pH via fermentation or addition of an acidulant could serve as an antimicrobial process by making the finished product unsuitable for *L. monocytogenes* growth. In this environment, microorganisms can be subtly damaged and while trying to repair their homeostatic mechanism to overcome the hostile environment, they begin to exhaust their energy reserves and die if they become metabolically depleted.

**Table 2:** Occurrence studies of *L. monocytogenes* in RTE meat products.

Country/Area	Sample Type	*n*	Prevalence	Reference
**Cooked Meat Products**				
New Zealand	Prepackaged pâté	300	0.33%	[16]
	Packaged cooked ham	104	0%	[16]
New Zealand	Cooked ham	301	3.3%	[17]
European Union	Heat treated RTE meat products	3530	2.07%	[18]
Belgium	Cooked meat products	3405	4.9%	[19]
Belgium	Cooked meat products	639	1.1%	[20]
Sweden	Cooked meat products	507	1.2%	[21]
United Kingdom	Cooked and sliced cold meat	2894	2.07%	[22]
	Cooked and sliced pâté	1184	1.86%	[22]
Spain	Pâté	182	5.4%	[23]
Spain	Sliced cooked meat products	68	7.3%	[24]
Spain	Vacuum-packed deli meat products	420	5.48%	[25]
	Vacuum-packed pâté	120	0.8%	[25]
Spain	Sliced cooked meat products	396	8.8%	[26]
Spain	Cooked products	35	5.71%	*This study*
**Raw-Cured Meat Products**				
Brazil	Salami	81	7.41%	[27]
Brazil	Sliced salami	45	6.7%	[28]
Turkey	Fermented sausages	300	11.7%	[29]
France	Dried sausages	30	10%	[30]
Italy	Fermented sausages	237	15.2%	[31]
Spain	Raw-cured sausages	72	30.55%	[32]
Spain	Raw-cured sausages	102	10.78%	[33]
Spain	Raw-cured sausages	19	15.8%	[34]
Spain	Cured meat products	345	6.7%	[26]
Spain	Raw-cured meat products	57	5.26%	*This study*
**Dry-Cured Salted Meat Products**				
Italy	Dry-cured and salted ham	490	4.1%	[35]
	Deboned ham	708	2%	[35]
Spain	Dry-cured salted products	37	2.70%	*This study*

RTE foods are considered an important food-borne source of human *L. monocytogenes* infections in the EU. The risk for human health arises from exposure to *L. monocytogenes* in such foods and in particular foods containing *L. monocytogenes* exceeding the level of 100 cfu/g. In this survey, only one sample of raw-cured sausage contained this pathogen at 190 cfu/g at half shelf-life, a level exceeding the food safety limit indicated by Commission Regulation (EC) No 2073/2005 on microbiological criteria for foodstuffs.

The growth characteristics of *L. monocytogenes* enable it to survive on equipment, in drains and on floors in cool and high humidity conditions, which makes it a highly persistent microorganism with repeated isolation in the food industry environment [36]. Additionally, RTE foods have a recognized potential for contamination with *L. monocytogenes*, either primary (by contaminated raw meat) or by cross-contamination from contaminated surfaces [37,38]. In food processing industries, there are many factors that influence the survival of microorganisms. Among these, processing machines are very important, especially those with complex designs which are difficult to clean [39].

According to a recent review by Meloni [1], the level of contamination by *L. monocytogenes* in food and non-food contact surfaces in meat processing environment ranges between 17%–50% and 11%–25%, respectively. The present study showed that 22.72% of work surfaces tested positive for *L.* *monocytogenes*. Gounadaki *et al.* [40] found 11.7% positives from a sample set of 34 zones of grinders, mixers, stuffing machines, knives, tables and cold rooms. Another study showed a lower incidence of *L. monocytogenes*, 1 positive sample in a stuffing machine from 20 samples taken in a cured sausage producing industry [41]. These results are far below those recorded by Salvat *et al.* [42], which showed 68.0% of samples to be positive for the presence of *L. monocytogenes* in the area of raw product and 33.0% in the area of finished products, for a set of 220 environmental swab samples from delicatessen industries after an outbreak of listeriosis in France. Similarly to the present study, Lundén *et al.* [43] found that food processing machines, such as slicers, spiral freezers, packaging machines and conveyor belts are often contaminated with *L. monocytogenes*. In Ireland the prevalence of *L. monocytogenes* on slicing machines used for cooked RTE meat products at retail level was found to be 0.23% [44]. In another study a high incidence (100%) of *Listeria* spp. was cited in swab samples from tabletops in a butchers shop in Nigeria [45]. Incidences closer to those obtained in current study include those found by Lakićević *et al.* [46], who showed that of 141 swab samples obtained in a restaurant in Belgrade (Serbia) 16.3% were positive for the presence of *Listeria* spp., the floors and drains being the most contaminated.

Stainless steel is the material of choice for equipment and utensil surfaces that come into direct contact with food because of its mechanical strength, resistance to corrosion, longevity and ease of manufacture. Although this surface may seem smooth, it is scored with many channels and holes, produced by polishing, within which microorganisms can anchor, making them difficult to eliminate. In addition, it is moderately hydrophilic with a negative surface charge and bacteria adhere to it easily and irreversibly [47]. Also commonly used are cutting boards made of plastics, such as polyethylene and nylon. They have the advantage of being easy to clean, but although they have non-porous surfaces, bacteria and food debris can become trapped in fissures. Food residues and bacteria are not easy to remove through manual washing, meaning these cutting boards become potential sources of pathogens.

Therefore, among the measures taken to avoid *L. monocytogenes* cross-contamination in the industries, particular attention must be paid to the cleaning and disinfection of the machines and surfaces in the processing plants. Machines should have a hygienic design allowing for proper cleaning, making them easy to dismantle and clean correctly. In addition, the processing line must be perfectly organized with the raw product area being well separated from the zone for finished products [43]. Good manufacturing practices, appropriate cleaning, sanitation and hygiene programs and effective temperature control throughout the food production, distribution and storage chain are required for prevention of contamination or inhibition of growth of *L. monocytogenes* to levels exceeding 100 cfu/g in foods that may pose a food safety risk.

## 5. Conclusions

This survey investigated the occurrence of *L. monocytogenes* in RTE meat products at manufacturing and retail, as well as in environmental samples from meat processing plants. The prevalence of *L. monocytogenes*-contaminated RTE meat products at the time of sampling was between 17.14 and 36.84%, while at the end of shelf-life ranged from 2.70 to 5.71 %. The proportion of RTE meat products exceeding the food safety limit of 100 cfu/g at the end of shelf-life was 0.78 %. *L. monocytogenes* was detected in 22.72% food contact surface samples from meat processing plants. Good manufacturing practices, appropriate cleaning, sanitation and hygiene programs and effective temperature control throughout the food production, distribution and storage chain are required for prevention of contamination or inhibition of growth of *L. monocytogenes* in foods.
